# ResNet-SE-CBAM Siamese Networks for Few-Shot and Imbalanced PCB Defect Classification

**DOI:** 10.3390/s25134233

**Published:** 2025-07-07

**Authors:** Chao-Hsiang Hsiao, Huan-Che Su, Yin-Tien Wang, Min-Jie Hsu, Chen-Chien Hsu

**Affiliations:** 1 Department of Computer Science and Information Engineering, Tamkang University, New Taipei City 251301, Taiwan; 164310@o365.tku.edu.tw; 2 Department of Mechanical and Electro-Mechanical Engineering, Tamkang University, New Taipei City 251301, Taiwan; 410350267@o365.tku.edu.tw; 3 Department of Artificial Intelligence, Tamkang University, New Taipei City 251301, Taiwan; 165910@o365.tku.edu.tw; 4 Innodisk Corporation, New Taipei City 221006, Taiwan; 5 Department of Electrical Engineering, National Taiwan Normal University, Taipei 10601, Taiwan; jhsu@ntnu.edu.tw

**Keywords:** defect detection, imbalanced datasets, few-shot learning, Siamese networks, Automatic Optical Inspection

## Abstract

Defect detection in mass production lines often involves small and imbalanced datasets, necessitating the use of few-shot learning methods. Traditional deep learning-based approaches typically rely on large datasets, limiting their applicability in real-world scenarios. This study explores few-shot learning models for detecting product defects using limited data, enhancing model generalization and stability. Unlike previous deep learning models that require extensive datasets, our approach effectively performs defect detection with minimal data. We propose a Siamese network that integrates Residual blocks, Squeeze and Excitation blocks, and Convolution Block Attention Modules (ResNet-SE-CBAM Siamese network) for feature extraction, optimized through triplet loss for embedding learning. The ResNet-SE-CBAM Siamese network incorporates two primary features: attention mechanisms and metric learning. The recently developed attention mechanisms enhance the convolutional neural network operations and significantly improve feature extraction performance. Meanwhile, metric learning allows for the addition or removal of feature classes without the need to retrain the model, improving its applicability in industrial production lines with limited defect samples. To further improve training efficiency with imbalanced datasets, we introduce a sample selection method based on the Structural Similarity Index Measure (SSIM). Additionally, a high defect rate training strategy is utilized to reduce the False Negative Rate (FNR) and ensure no missed defect detections. At the classification stage, a K-Nearest Neighbor (KNN) classifier is employed to mitigate overfitting risks and enhance stability in few-shot conditions. The experimental results demonstrate that with a good-to-defect ratio of 20:40, the proposed system achieves a classification accuracy of 94% and an FNR of 2%. Furthermore, when the number of defective samples increases to 80, the system achieves zero false negatives (FNR = 0%). The proposed metric learning approach outperforms traditional deep learning models, such as parametric-based YOLO series models in defect detection, achieving higher accuracy and lower miss rates, highlighting its potential for high-reliability industrial deployment.

## 1. Introduction

An Automatic Optical Inspection (AOI) system is typically employed in a printed circuit board (PCB) production line to detect component defects. During the inspection process, even a single undetected defect—such as a microscopic solder crack or an oxidized pad—can lead to extensive product recalls, costly customer returns, and significant damage to the brand [1]. In such critical environments, achieving zero missed detections, or a zero False Negative Rate (FNR), is not a luxury but a necessity.

Most AOI systems have started integrating deep learning technologies to enhance defect detection capabilities. Conventional deep learning methods, such as the YOLO series architectures, demonstrate strong performance when trained on large and balanced datasets [2]. However, in real-world production environments, normal samples vastly outnumber defective ones, resulting in datasets with a small number of defective features and highly imbalanced normal–defective samples [3]. Under these imbalanced conditions, deep learning models like YOLO often struggle to maintain stable detection rates [4,5]. Particularly when dealing with extremely limited defective samples from actual production lines, issues such as overfitting and a high FNR frequently emerge.

To address these challenges, this study proposes a PCB defect detection system specifically designed for few-shot and imbalanced scenarios. A real-world PCB production image dataset provided by Innodisk Corporation was utilized [6], covering common defect types such as solder cracks, oxidation, and pin misalignments. Instead of using complex deep learning models like the YOLO series architecture, we propose to build a small-sized, metric-based Siamese network for detecting defects in small and imbalanced datasets. The proposed Siamese network integrates Residual blocks (ResNet) [7], Squeeze and Excitation blocks (SE) [8], and Convolution Block Attention Modules (CBAM) [9] (namely ResNet-SE-CBAM-based Siamese network) for feature extraction. It is optimized through triplet loss [10] to construct an embedding space where intra-class distances are minimized and inter-class distances are maximized. In real-world production line deployment, the metric-based Siamese network allows for the addition or removal of classes without the need to retrain the model [11]. To further enhance training under limited data conditions, a Structural Similarity Index Measure (SSIM)-based sample selection technique [12] was introduced, improving both sample diversity and model generalization. Additionally, a high defect rate training strategy was utilized to reduce the FNR and ensure no missed defect detections. At the classification stage, a K-Nearest Neighbor (KNN) classifier [13] was incorporated to directly operate in the feature space, mitigating overfitting risks and improving classification robustness in few-shot environments.

We conducted experimental comparisons, with the results highlighting the effectiveness of the proposed approach in addressing the practical challenges of defect detection in real-world PCB production lines, even under highly constrained data conditions.

The main contributions of this study are summarized as follows:

- A PCB defect detection framework, ResNet-SE-CBAM Siamese network, was developed for feature extraction on datasets with limited defects. Optimized with triplet loss embedding learning [10], it is tailored for few-shot and imbalanced data environments.
- A Siamese network incorporating novel attention mechanisms was proposed to enhance defect detection performance. Meanwhile, its metric learning capability allows for the addition or removal of feature classes without the need to retrain the model, improving its applicability in industrial production lines with limited defect samples.
- An SSIM-based sample selection technique [12] was introduced to select diverse and representative training samples, enhancing learning stability and model generalization. Additionally, a high defect rate training strategy is utilized to reduce the FNR and ensure no missed defect detections.
- Comprehensive experiments and comparisons have been conducted to validate the performance of the proposed systems and algorithms in terms of defect detection capability, missed defect detection prevention, metric learning efficiency, and practical applicability for high-reliability production lines.

## 2. Related Work

### 2.1. PCB Defect Detection

As the precision and production speed of electronic products continue to advance, the importance of PCB defect detection has grown significantly. Traditional AOI systems primarily relied on handcrafted features and machine learning classifiers for defect identification, including methods such as template matching, edge detection, and morphological operations [14]. While these approaches can achieve reasonable performance under specific conditions, they generally perform poorly when encountering highly variable or unexpected defect types.

In recent years, the rise in convolutional neural networks (CNNs) has accelerated the evolution of PCB defect detection toward deep learning-based frameworks [15,16]. Studies such as those by Bhattacharya and Cloutier [17] proposed CNN-based defect classification systems, while Lan et al. [18] demonstrated the application of YOLOv3 for rapid surface defect detection on PCBs. These works highlight the potential of deep learning in improving defect detection rates. However, such methods typically rely on large-scale, balanced datasets, and often suffer from overfitting and loss of detection stability under few-shot or highly imbalanced data scenarios [4,5].

To address these limitations, researchers have explored techniques such as feature recombination and data augmentation to adapt to few-shot environments [19]. Generative adversarial networks (GANs) are commonly used to synthesize minority-class samples for imbalanced datasets. For example, Azad et al. [20] employ a conditional GAN to generate additional delamination defect images under limited data conditions. Nevertheless, practical deployment on production lines still faces challenges related to data sparsity and limited defect diversity. Motivated by these issues, this study designs a defect detection system based on few-shot feature learning and sample selection strategies, aiming for better adaptability to real-world production environments.

### 2.2. Few-Shot Learning

Few-shot learning aims to achieve effective model training with only a small number of labeled samples. Existing methods mainly fall into two categories: metric learning and meta-learning [21]. Among them, Siamese networks [22] and Triplet Networks [23] are representative metric learning approaches, which train models to learn the distance relationships between samples for similarity-based classification. Few-shot learning has gained increasing attention in anomaly and defect detection fields. For example, Wang et al. [24] utilized few-shot metric learning for surface defect detection, while Lin et al. [25] proposed a meta-learning framework combined with Prototype Networks for defect recognition under high intra-class variability. However, many existing few-shot learning methods assume that intra-class variation is minimal and that data is relatively clean—assumptions that do not align well with the high heterogeneity observed in real-world PCB defect samples [26].

Building on the Siamese network architecture, this study incorporates triplet loss [10] for distance optimization in the feature space. It mitigates the risk of overfitting and feature collapse, ensuring robust classification performance under few-shot, imbalanced conditions. To ensure a compact model size and high generalization ability even on small datasets, VGG ConvNet [27] is chosen as the main block of the network. Recently, attention mechanisms have been integrated into networks to enhance model performance in feature extraction. Notable examples include the SE block [8], CBAM block [9], and Coordinate Attention (CA) [28]. These modules compute channel and spatial attention, as well as positional information, to augment the representations of objects of interest. For multi-class defect detection and classification in real-world production lines, the metric-based Siamese network allows for the addition or removal of classes without the need to retrain the model [11]. In contrast, the YOLO series architecture uses parametric learning, requiring the model to be retrained whenever a new class is added.

### 2.3. Imbalanced Learning

Data imbalance remains a pervasive and critical challenge in real-world classification tasks [29]. Classical approaches to handling imbalance include under-sampling [30], over-sampling techniques such as SMOTE [31], and designing cost-sensitive loss functions, such as focal loss [32]. In the context of small-sample imbalance problems, recent studies have explored feature-space level solutions, such as class-balanced loss [33] and meta-weight networks [34], which aim to reweight or redistribute training samples dynamically. However, when directly applied to PCB defect detection, standard imbalance handling techniques often underperform due to the inherently high heterogeneity of defect samples [35]. GANs are also commonly used to synthesize minority-class samples for imbalanced datasets, generating additional delamination defect images under limited data conditions [20]. While such augmentation can improve apparent accuracy when class ratios are extreme, it often introduces artifacts and requires complex adversarial training.

For the small and imbalanced dataset, this study utilizes an SSIM-based [12] sample selection mechanism to preserve data representativeness and diversity during the under-sampling process. This strategy is combined with a high defect rate training strategy to reduce the FNR and ensure no missed defect detections.

## 3. Methodology

This section describes the design process of the proposed PCB defect detection system, including the overall system architecture, data preprocessing, feature extraction and training strategies, as well as classifier design and postprocessing mechanisms. The system was designed to maintain a balance between defect detection sensitivity and classification performance, even under few-shot and highly imbalanced data conditions.

### 3.1. System Architecture

**Figure 1:**
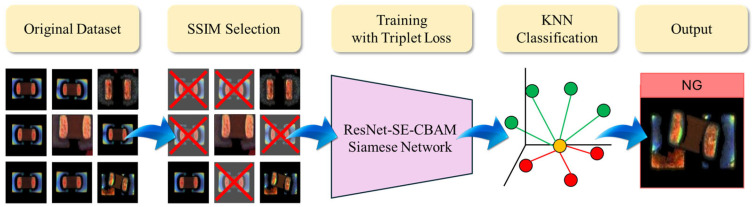
Overall training flowchart of the PCB defect detection system.

The architecture of the proposed PCB defect detection system is illustrated in Figure 1. The system comprises four main stages: image preprocessing, feature extraction and distance optimization, classification decision, and postprocessing. Initially, input images undergo normalization and data augmentation, followed by representative sample selection based on the SSIM to construct the training dataset. Feature extraction is performed using a Siamese network based on the ResNet-SE-CBAM architecture, and the inter-class distance structure within the embedding space is optimized through triplet loss. Finaly, classification is conducted in the embedding space using a KNN classifier, with feature standardization and principal component analysis (PCA) rotation applied to enhance classification stability and reduce the FNR.

### 3.2. Data Preprocessing and Sample Selection

To address the scarcity of defective samples and data imbalance inherent in PCB production lines, this study first applied normalization and image transformation to the input images. All images were resized to a uniform resolution of 224 × 224 pixels, and contrast normalization was performed to reduce variability caused by differences in imaging conditions. During training, random image transformation techniques were further applied, including random rotations (±10°), horizontal and vertical flips, and contrast variations (±15%), to simulate natural disturbances and variability encountered in real production environments.

For sample selection, this study employed the SSIM to filter the data, aiming to maintain both diversity and boundary representativeness. Among the normal samples, combinations exhibiting greater structural differences were prioritized to enhance the internal diversity of the training set. In addition, normal samples most similar to defective samples were selected to strengthen the classifier’s ability to discriminate near category boundaries. For defective samples, those maximizing structural diversity were selected to ensure a wide range of defect features were captured, thereby improving the model’s generalization capability.

**Figure 2:**
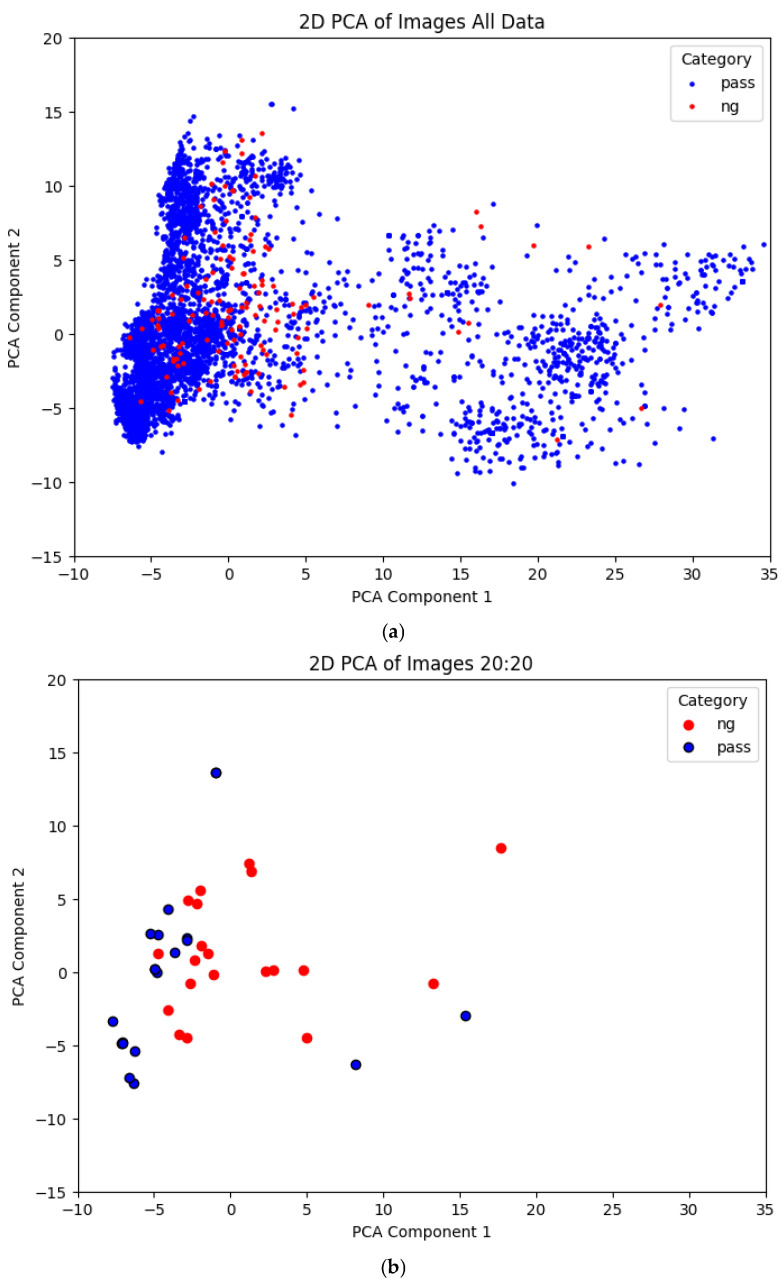
Two-dimensional PCA visualization of the dataset: (**a**) before and (**b**) after SSIM-based sample selection.

Figure 2 illustrates the image sample distribution before and after SSIM-based sample selection. The samples were transformed into two-dimensional data using PCA for visualization. As shown in Figure 2a, the original imbalanced dataset contained a large and densely distributed number of 900 normal (pass) samples and a smaller set of 150 defective (NG) samples. In contrast, Figure 2b shows that after SSIM filtering, although the total number of samples was reduced to a 20:20 pass to NG ratio, the overall distribution trend remained consistent with the original dataset. This demonstrates that the selection strategy effectively preserved the intrinsic characteristics of the data.

Through the above preprocessing and sample selection strategies, this study established a representative and highly discriminative dataset at the early stage of training, laying a solid foundation for subsequent feature learning and classification performance.

### 3.3. ResNet-SE-CBAM Siamese Feature Extraction Architecture

**Figure 3:**
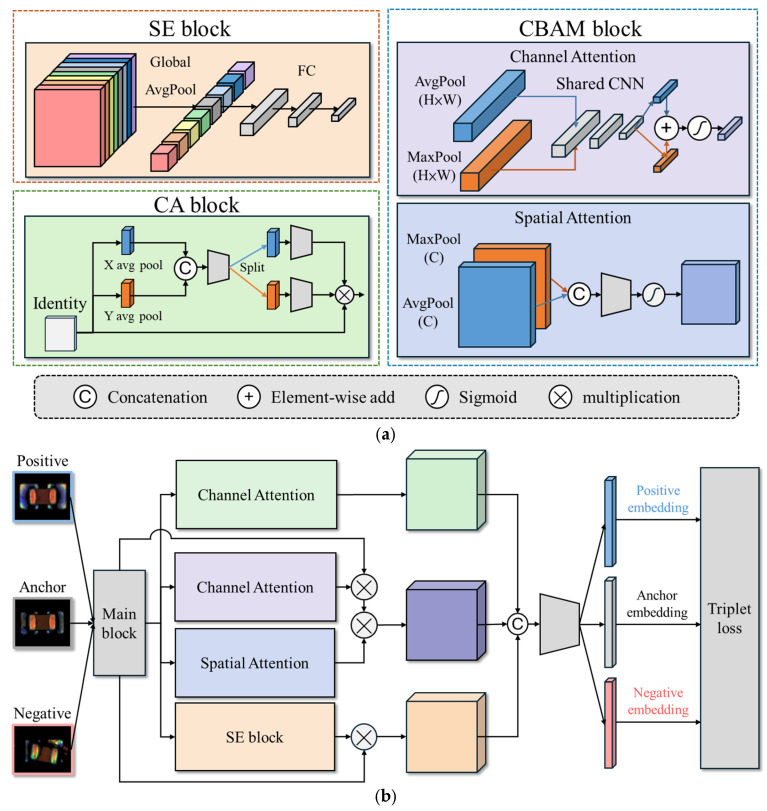
The Siamese network architecture. (**a**) Attention architectures include the SE, CBAM, and CA blocks; (**b**) The integrated Siamese network.

**Figure 4:**
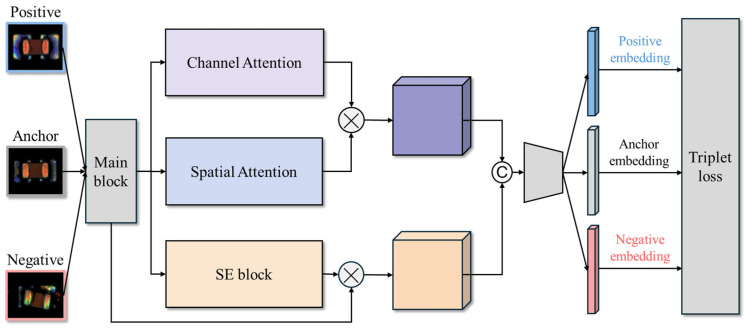
The ResNet-SE-CBAM Siamese network architecture.

The core feature extraction model proposed in this study is a Siamese network integrated with attention mechanisms. Prominent attention architectures include the SE block [8], CBAM block [9], and CA block [28], as illustrated in Figure 3a. The Siamese network architecture integrates four feature enhancement mechanisms, as depicted in Figure 3b. First, ResNet-18 [7] is employed as the backbone network, utilizing residual connections to mitigate the vanishing gradient problem, accelerate model convergence, and improve stability in deep networks. Second, a SENet module [8] is attached after each Residual block, which models the inter-channel relationships and adaptively recalibrates the importance of each feature channel, effectively strengthening critical feature representations. Third, a CBAM is incorporated, combining channel attention and spatial attention submodules using convolutions to guide the network’s focus toward important regions within the image, such as defect features [9]. Additionally, the CA block can be added to encode feature maps into direction-aware and position-sensitive attention maps, which can be complementarily applied to augment the representations of objects of interest [28]. These modules compute channel and spatial attention, as well as positional information, to augment the representations of objects of interest. However, as shown in the experimental results in Section 4.3, the addition of the CA block increases the FNR and results in more missed defect detections. Therefore, we will construct the core feature extraction model using only three feature enhancement mechanisms: the ResNet-SE-CBAM Siamese network, as depicted in Figure 4.

The overall architecture in Figure 4 consists of two sub-networks sharing the same weights, designed to learn similarity-based feature representations between image pairs. Each training sample comprises three images: an Anchor, a Positive (same-class sample), and a Negative (different-class sample). The model is trained using triplet loss, with the objective of minimizing the distance between samples of the same class while maximizing the distance between samples of different classes, thereby forming highly discriminative embeddings in the feature space.

After feature extraction, each input image is mapped by the Siamese network into a 64-dimensional embedding vector. To learn an effective feature space, triplet loss [10] is adopted as the loss function, mathematically defined as follows:

(1) $$L_{\mathrm{triplet}}=\max\left({\left\|f\left(x_{a}\right)-f\left(x_{p}\right)\right\|}_{2}^{2}-{\left\|f\left(x_{a}\right)-f\left(x_{n}\right)\right\|}_{2}^{2}+\alpha ,0\right)$$

where $f\left(\cdot \right)$ denotes the feature embedding function, and $x_{a}$, $x_{p}$ and $x_{n}$ represent the Anchor, Positive, and Negative samples, respectively. The parameter α is a margin value, set to 0.2 in this study, ensuring that the distance between different-class samples exceeds that between same-class samples by at least the specified margin.

During the training process, 512 triplet sets are randomly generated in each epoch, and semi-hard mining is employed to select sample pairs with moderate difficulty for training. The Adam optimizer is used with an initial learning rate of $1\times 10^{-4}$, and the learning rate is further adjusted using a Cosine Annealing schedule to promote stable convergence. The training is conducted for up to 100 epochs, with an Early Stopping strategy applied: training is terminated early if the validation loss does not improve for 10 consecutive epochs. All training procedures are conducted on an NVIDIA RTX 4090 GPU platform, with each complete training session taking approximately 45 min.

Through the feature extraction network design and the metric learning training strategy described above, a highly discriminative embedding space can be established under few-shot and imbalanced data conditions, providing a stable and effective foundation for the subsequent classification stage.

### 3.4. Classifier Design and Postprocessing

After feature extraction and embedding space optimization, KNN [13] was adopted as the final classifier to address the classification challenges under few-shot and imbalanced data conditions. KNN makes decisions based on the Euclidean distance in the feature space, requiring no parameter training and imposing minimal assumptions on the data distribution, making it particularly suitable for applications with sparse and limited samples.

Traditional classifiers, such as multilayer perceptron (MLP) or support vector machines (SVM), are prone to overfitting when the sample size is small, resulting in poor generalization performance [36]. This problem is further exacerbated when defective samples are extremely scarce and exhibit high intra-class variation, making it difficult for complex classifiers to learn stable decision boundaries. In contrast, KNN, as a non-parametric, instance-based classification method, directly infers predictions based on sample-to-sample distances, exhibiting better stability and interpretability under few-shot conditions [37].

To improve the stability of distance calculations in the feature space, Z-score normalization was first applied to all embedding vectors to eliminate scale differences across feature dimensions [38]. Subsequently, PCA was performed to rotate the feature axes. PCA orthogonally transforms the features to reduce inter-feature correlation, thereby enhancing the representativeness and stability of Euclidean distance measurements [39]. It is important to note that in this study, only feature rotation was performed without dimensionality reduction, preserving the original 64-dimensional feature information to maintain classification performance.

**Figure 5:**
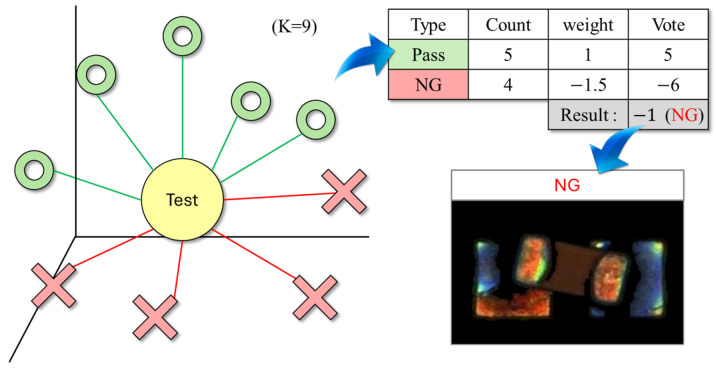
Distance and class-weighted voting illustration for KNN-based defect detection.

During the classification stage, the embedding vector of each test sample is compared with all training samples by computing the Euclidean distance after PCA rotation. The K-Nearest Neighbor is then selected, and a weighted voting mechanism is applied based on their class labels. To enhance the recall of defective samples, a class weight adjustment strategy was introduced: the voting weight of defective samples was set to be 1.5 times that of normal samples. This design ensures that even if defective samples are underrepresented among the neighbors, they can gain an advantage in the voting process through weighted influence, thereby reducing the risk of false negatives due to class imbalance. The overall concept is illustrated in Figure 5, where the test sample is positioned at the center of the feature space, surrounded by both normal and defective samples. Through distance computation and weighted voting, the classifier is able to sensitively detect defective samples, even when they are fewer in number.

Through the classifier design described above, this study achieves a balance between classification stability and defect detection sensitivity under few-shot and imbalanced conditions. Unlike conventional approaches that rely heavily on large datasets or complex hyperparameter tuning, the proposed embedding space optimization combined with a simple weighted KNN classifier enables stable operation under limited sample conditions, achieving outstanding defect detection performance across datasets with varying defect-to-good ratios.

Notably, when the proportion of defective samples was increased (e.g., 80:20 configuration), the system consistently maintained a zero-miss detection standard (FNR = 0%), demonstrating both the flexibility and reliability of the proposed design and its strong potential for direct deployment in real-world PCB defect detection tasks.

## 4. Experiments and Results

This section describes the experimental design, evaluation metrics, and results of the proposed defect detection system. Multiple comparative and ablation studies are conducted to validate the impact of different modules and parameter settings on overall system performance. In addition, the stability and generalization capability of the model are evaluated under varying data ratios and limited sample conditions.

### 4.1. Dataset and Experimental Settings

A real-world production image dataset provided by Innodisk Corporation [6] was used in this study. The dataset consists of high-resolution close-up images of electronic components, labeled into two categories: “defective” and “normal.” Defect types include common issues such as solder cracks, oxidation, and pin misalignments. The dataset contains a total of 1050 images, comprising 150 defective samples and 900 normal samples, resulting in an approximate defect-to-good ratio of 1:6, as summarized in Table 1. To promote reproducibility and open research, we published the dataset on Hugging Face [6].

In these experiments, the high defect rate training strategy was employed to better accommodate the diverse and sparse conditions of PCB defect detection tasks. The training dataset was fixed at 20 normal samples, combined with varying numbers of defective samples. The number of defective samples was set to 20, 40, 60, 80, and 100, corresponding to defect-to-good ratios of 1:1, 2:1, 3:1, 4:1, and 5:1, respectively. This setup was used to investigate the model’s recognition capability and stability under different sample size conditions, as detailed in Table 2.

All image data were normalized and uniformly resized to 224 × 224 pixels to standardize the input format and facilitate model convergence. During the training phase, random image transformation techniques, including rotations (±10°), horizontal and vertical flipping, and contrast perturbations, were applied to enhance feature diversity and reduce the risk of overfitting.

In addition, triplet samples were dynamically generated in each training epoch, forming Anchor, Positive, And Negative sets. Triplet loss was employed to optimize the embedding space, encouraging the model to minimize intra-class distances and maximize inter-class distances, thereby strengthening the discriminative power of the learned features.

### 4.2. Evaluation Metrics

Given that the PCB defect dataset used in this study exhibits a highly imbalanced distribution, with a significantly larger number of normal samples compared to defective samples and considering that real-world production environments have extremely low tolerance for missed defects (i.e., misclassifying defective samples as normal), three evaluation metrics were selected: accuracy, FNR, and Matthews Correlation Coefficient (MCC).

Accuracy measures the overall proportion of correct predictions made by the model and is defined as

(2) $$\mathrm{Accuracy}=\frac{TP+TN}{TP+TN+FP+FN}$$

where TP denotes the number of defective samples correctly classified as defective, TN represents the number of normal samples correctly classified as normal, FP refers to normal samples misclassified as defective, and FN indicates defective samples misclassified as normal. Although accuracy provides an overall view of classification capability, relying solely on accuracy under extreme class imbalance may lead to misleading interpretations; thus, it is used only as an additional evaluation metric for reference in this study.

The FNR quantifies the proportion of defective samples incorrectly classified as normal and is defined as

(3) $$\mathrm{FNR}=\frac{FN}{FN+TP}$$

FNR serves as the most critical evaluation metric in this study. In practical production scenarios, misclassifying a defective product as normal can lead to severe consequences, including product failure, costly repairs, or customer complaints. Therefore, the system must prioritize minimizing the FNR, even at the expense of increasing the false positive rate (FPR) if necessary.

Considering the extreme imbalance of the dataset, the MCC was also adopted as a supplementary metric to evaluate the overall classification performance. MCC takes into account all four classification outcomes—TP, TN, FP, and FN—and provides a more objective assessment of model performance under imbalanced conditions. MCC is calculated as

(4) $$\mathrm{MCC}=\frac{TP\times TN-FP\times FN}{\sqrt{\left(TP+FP\right)\left(TP+FN\right)\left(TN+FP\right)\left(TN+FN\right)}}$$

The MCC value ranges from [−1, 1], where a value close to 1 indicates perfect classification, 0 indicates performance equivalent to random guessing, and negative values suggest an inverse relationship between predictions and true labels. Given the small number and high difficulty of detecting defective samples, relying solely on accuracy can overestimate system performance. Incorporating MCC enables a more comprehensive and balanced evaluation of the model’s true defect recognition capability.

In summary, this study uses FNR as the primary evaluation metric, supplemented by MCC and accuracy, to ensure that the constructed defect detection system maintains a low missed detection rate and strong overall stability, even under extremely imbalanced data conditions.

### 4.3. Ablation Study of Siamese Architectures

To investigate the impact of feature extraction architectures on classification performance, four different Siamese network architectures were compared under the setting of 20 normal samples and 40 defective samples. All experiments consistently used triplet loss as the training objective and employed KNN as the backend classifier to eliminate any influence from classifier differences. The architecture evaluated in this study includes eight Siamese networks, as depicted in Table 3. The table summarizes the performance of each architecture in terms of accuracy (ACC), FNR, and MCC.

The results in Table 3 indicate that the ResNet-Siamese architecture slightly enhances defect recognition stability compared to the basic CNN–Siamese architecture, achieving an accuracy of 90% and an MCC of 0.81. Incorporating attention mechanisms through ResNet-SE and ResNet-CA modules results in zero FNR, while maintaining accuracies of 90% and 92%, respectively, and slightly increasing MCC to 0.81 and 0.85. However, the ResNet-SE and ResNet-CA modules significantly increase FNR when trained with other PCB components, as shown in Section 4.9. Incorporating attention mechanisms via ResNet-SE-CBAM results in an acceptable FNR of 2%, whereas the ResNet-SE-CBAM-CA module exhibits a high FNR of 4%. The ResNet-SE-CBAM module performs well when training with other PCB components as demonstrated in Section 4.9. It also shows strong robustness in defect detection, confirming that the addition of spatial attention enables the model to more effectively localize subtle defect features. Overall, the feature extraction architecture design significantly impacts defect detection performance under few-shot conditions. Specifically, the introduction of channel and spatial attention mechanisms effectively enhances the model’s focus on critical regions, thereby improving overall classification performance and reinforcing its reliability for real-world production line deployment.

### 4.4. Classifier Comparison

To further analyze the classifier selection impact on overall recognition performance, this section compares four different classifiers evaluated using the same embedding features. All features were extracted using the ResNet-SE-CBAM Siamese network, and the experimental setting was fixed at 20 normal samples and 40 defective samples to ensure fairness. The classifiers compared include KNN, SVM, XGBoost, and MLP.

The results in Table 4 indicate that the KNN and MLP classifiers achieved better performance in terms of both accuracy and MCC, with accuracies of 94% and 93% and MCCs of 0.88 and 0.83, respectively, suggesting strong classification stability when sufficient feature learning is achieved. However, MLP models typically require larger datasets for effective training, raising concerns about their stability and generalization capability in real-world production scenarios where sample sizes are extremely limited.

Although SVM also achieved an accuracy of 93%, its MCC was slightly lower than that of KNN and MLP, indicating limitations in learning robust decision boundaries under few-shot conditions. As for XGBoost, while it achieved a zero-miss rate (FNR = 0%), its overall accuracy was only 84% with an MCC of 0.72, suggesting unstable classification results and a tendency to misclassify normal samples.

**Figure 6:**
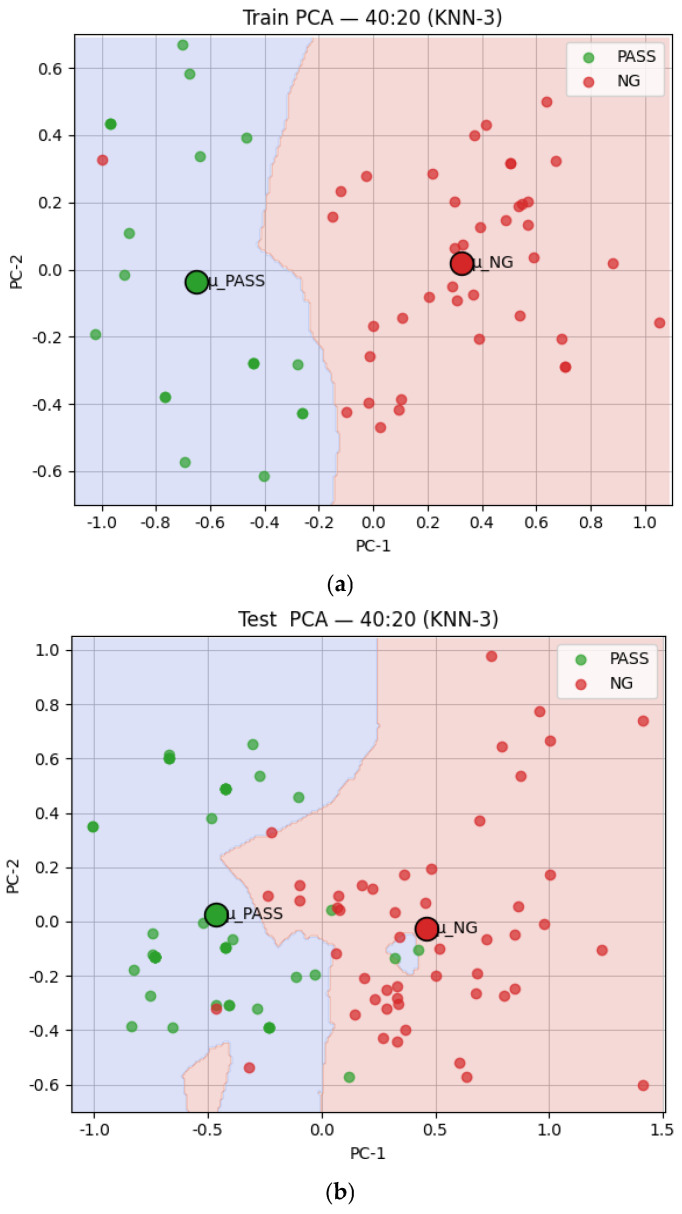
Two-dimensional visualization of KNN classification results. (**a**) The training results; (**b**) the test results.

Considering the balance between classification accuracy, defect detection capability, model stability, and practical deployment needs, this study ultimately selected KNN as the final classifier for the defect detection system to ensure a reliable, stable, and efficient solution for real-world production applications. Figure 6 depicts the KNN classification 2D visualization results. The training results with a 40:20 pass to NG ratio and the test results with a 50:50 pass to NG ratio are shown in Figure 6a and b, respectively.

### 4.5. Determining the Number of Neighbors

**Figure 7:**
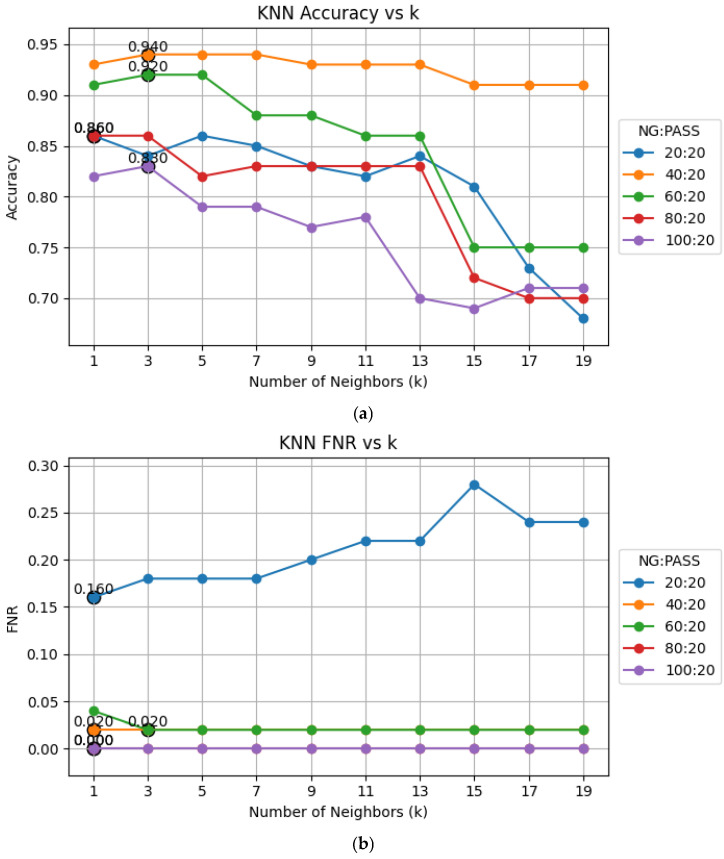

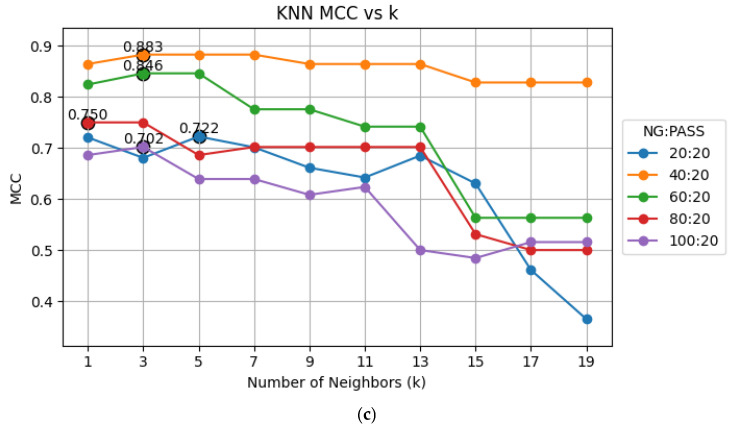
Performance plots against various K values: (**a**) accuracy; (**b**) FNR; (**c**) MCC.

The elbow method is used to determine the optimal number of neighbors for KNN classification by plotting accuracy, FNR, and MCC against various K values and identify the point of diminishing returns. The results, depicted in Figure 7, indicate that K = 3 has the best performance in terms of accuracy, FNR, and MCC. Therefore, we chose K = 3 for our experiments.

### 4.6. Testing with Different Defect Sample Ratios

In these experiments, the high defect rate training strategy was employed to better accommodate the PCB defect detection tasks’ diverse and sparse conditions. To investigate the impact of varying defective sample quantities on model performance, the number of normal samples was fixed at 20, while the number of defective samples was gradually increased to 20, 40, 60, 80, and 100. Training and testing were conducted under each setting. In all experiments, the ResNet-SE-CBAM Siamese network was used as the feature extractor, and KNN was employed as the classifier to maintain consistency in experimental settings.

The results in Table 5 show that increasing the number of defective samples from 20 to 40 significantly improved model performance, raising accuracy to 94%, reducing FNR to 2%, and increasing MCC to 0.88. A moderate increase in the number of defective samples clearly enhanced defect feature learning. When the number of defective samples increased to 80, the model achieved zero-miss detection (FNR = 0%), although the accuracy and MCC declined to 86% and 0.75, respectively. Defect discrimination and boundary stability further improved. At 100 defective samples (defective-to-good ratio of 5:1), both accuracy and MCC further declined, likely due to excessive class imbalance and insufficient normal samples. Overall, moderate increases in defective samples improved sensitivity and reduced missed detections. However, excessive imbalance degraded performance, highlighting the importance of careful data ratio planning.

### 4.7. Comparison with YOLO Series Models

To validate the performance of the proposed metric-based Siamese network architecture in defect detection tasks, comparisons were conducted under 1:1 and 1:2 good-to-defect sample ratios between the ResNet-SE-CBAM Siamese model combined with a KNN classifier and the parametric-based YOLO series models (YOLOv7, YOLOv7-tiny, YOLOv12-Nano, and YOLOv12-Small).

The results in Table 6 show that the proposed ResNet-SE-CBAM Siamese model achieved superior overall performance with a 1:2 pass to NG ratio, yielding the highest accuracy (94%) and MCC (0.88), and the lowest FNR (2%) among all evaluated models. While YOLOv7-tiny exhibited a low FNR (6%), its moderate accuracy and MCC (74% and 0.52, respectively) reflect a less balanced classification outcome. In contrast, YOLOv7 and YOLOv12 showed weaker performance with accuracies of 63% and 91%, and FNRs of 42% and 18%, respectively, suggesting poor sensitivity to subtle defect patterns. In summary, under balanced and imbalanced data conditions and scenarios demanding fine-grained defect recognition, the proposed attention-augmented Siamese network demonstrates marked advantages in classification stability and detection performance. These findings underscore its strong potential for deployment in real-world industrial inspection applications.

### 4.8. Testing on GAN-Based Image Data Augmentation

**Figure 8:**
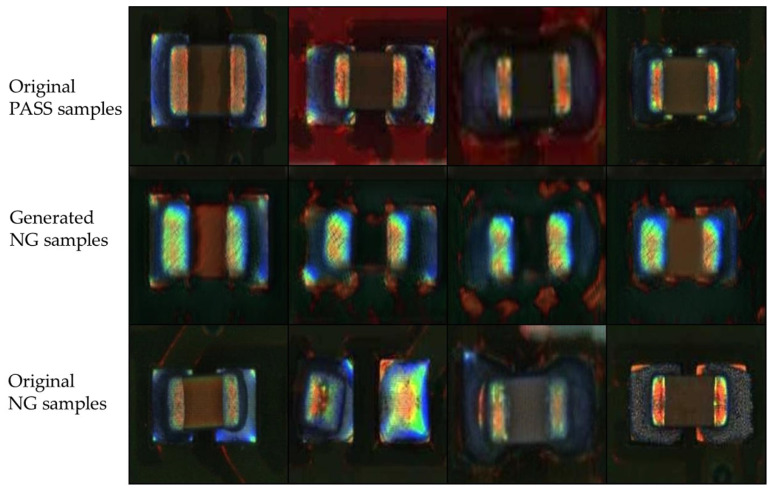
Original and GAN-generated image samples.

To address the issues with limited and imbalanced datasets, researchers have explored techniques such as GANs to synthesize and augment minority-class samples [20]. In this experiment, we utilized GANs to generate 1800 defect images based on 900 normal (pass) and 150 defect (NG) samples. Four of these original and generated images are shown in Figure 8. We further selected 200 generated images based on SSIM, choosing the generated samples most similar to the original 150 defective samples to augment the defect dataset. We retained the ResNet-SE-CBAM Siamese network with a high defect rate by adding the 200 GAN-generated defect images to the 40 defect data points. The results listed in Table 7 indicate that the dataset with GAN-generated defect images has weaker performance, with accuracy of 84%, FNR of 10%, and MCC of 0.68.

### 4.9. Metric Learning

Preventing test–training leakage is crucial when dealing with multiple types or classes of abnormal defects [40]. To further assess the generalizability and zero-shot capabilities of the proposed Siamese networks with multiple classes of abnormal defects, we conducted an experiment on metric learning for detecting different PCB components. Metric learning allows for the addition or removal of classes without the need to retrain the model. In this study, we trained and tested the Siamese network model separately for each class of datasets. For subsequent classes, we used zero-shot metric learning without retraining the model. This approach eliminates the risk of test–training leakage. In contrast, parametric-based deep learning models like YOLO train the model with all classes together, which poses a risk of test–training leakage during data augmentation, even if the data is initially split into training and testing sets.

**Figure 9:**
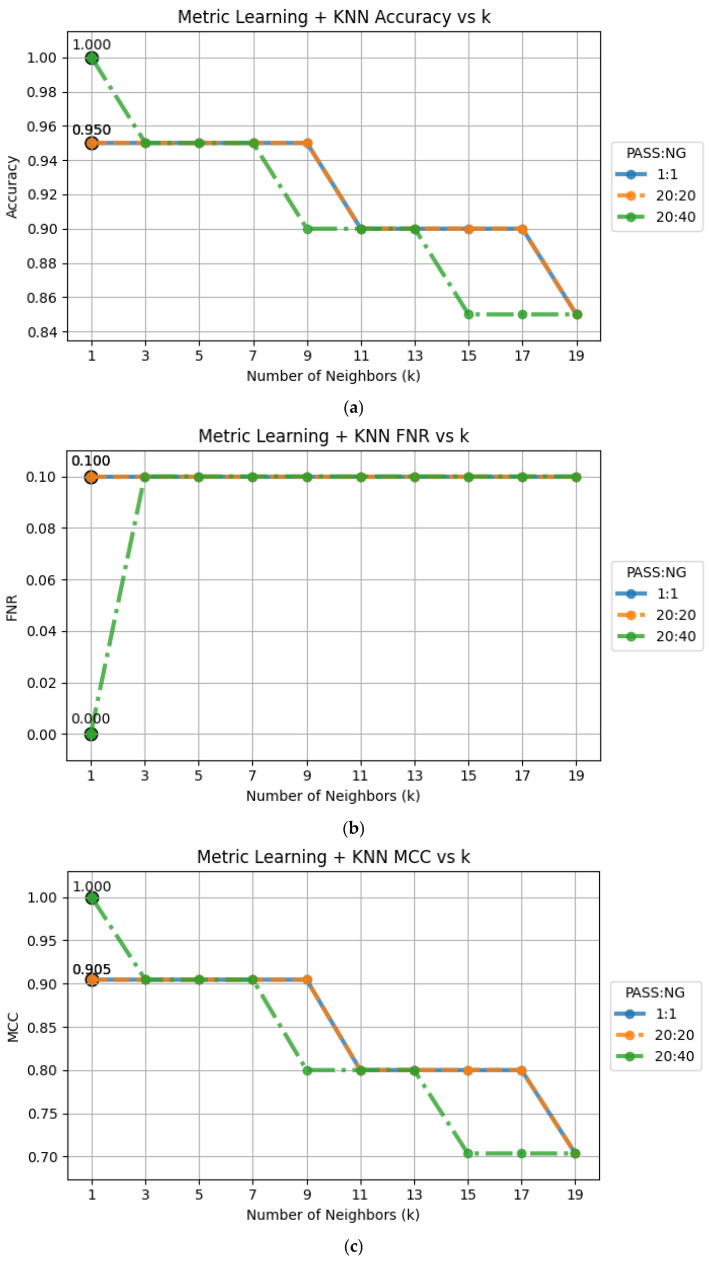
The zero-shot experimental results using 1:1, 20:20, and 40:20 resistor test datasets: (**a**) accuracy; (**b**) FNR; (**c**) MCC.

This experiment involved new resistor components, which were not included in any previous training or evaluation steps. Notably, all experiments in Section 4.1, Section 4.2, Section 4.3, Section 4.4, Section 4.5, Section 4.6, Section 4.7 and Section 4.8 were conducted exclusively on capacitor samples. In this experiment, we utilized the pre-trained ResNet-SE-CBAM model, which was pre-trained using a 20:40 pass to NG ratio capacitor dataset as described in Section 4.6. The zero-shot experiments were conducted using 1:1, 20:20, and 40:20 pass to NG ratio resistor test datasets (called golden samples or support sets). The accuracy results, FNR, and MCC against various K values are depicted in Figure 9. The results indicate that all three test datasets performed well in terms of accuracy (≥85%), FNR (≤10%), and MCC (≥0.70).

### 4.10. Comparison of Zero-Shot and Retraining Model

In this section, we conducted an experimental configuration to evaluate the metric learning capabilities of the Siamese architecture, specifically its ability to generate and compare meaningful embeddings without additional training. Two configuration settings were planned: trained setting and zero-shot setting. Under the trained setting, the model was fine-tuned using new resistor samples. In contrast, under the zero-shot setting, the same support set (golden samples) was provided, but the model weights remained unchanged—no retraining was performed. For test datasets, we employed 10 defective and 10 normal resistor images to simulate a new deployment scenario characterized by extremely limited labeled data. The results shown in Table 8 indicate that the ResNet-SE-CBAM Siamese model achieved strong performance even in the zero-shot setting, with accuracy and FNR comparable to the model trained on resistor samples.

**Figure 10:**
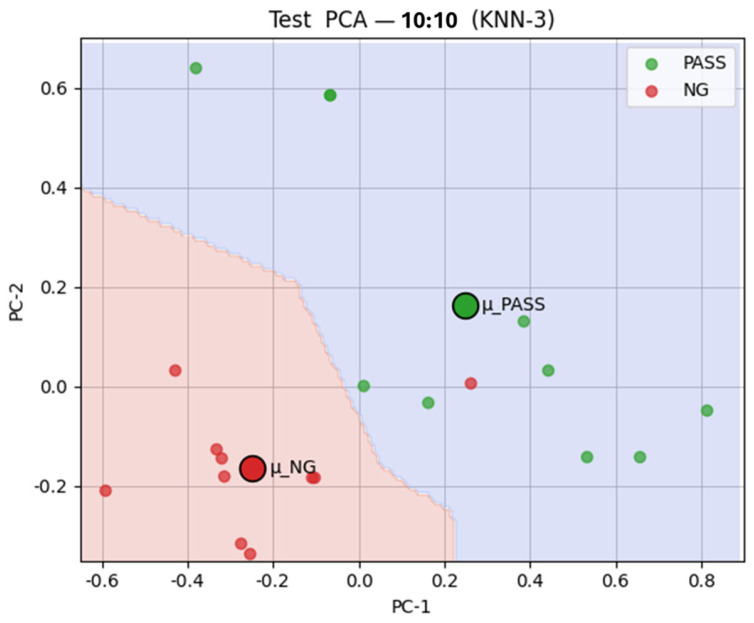
The 2D zero-shot classification visualization results for the ResNet-SE-CBAM model for a 10:10 test dataset.

These findings highlight two key strengths of the proposed framework: its cross-component generalizability, wherein a model trained on capacitors can effectively detect defects in resistors, and its zero-shot classification capability, demonstrating that the system can perform reliable matching and inference solely through learned similarity measures, without requiring further training. A zero-shot classification example of the ResNet-SE-CBAM model with 20:40 pre-trained similarity measures is depicted in Figure 10.

## 5. Conclusions

This study proposed a deep learning-based PCB defect detection system centered on a ResNet-SE-CBAM Siamese architecture to address the challenges of few-shot and imbalanced data environments. By integrating channel and spatial attention mechanisms, SSIM-based weighted sample selection, a high defect rate training strategy, and triplet loss feature space optimization, the system effectively enhanced defect feature recognition while maintaining overall classification stability.

The experimental results showed that under a 1:2 good-to-defect sample ratio (20:40), the ResNet-SE-CBAM Siamese model combined with a KNN classifier achieved a classification accuracy of 94% and a low FNR of only 2%. When the defect sample ratio was further increased to 1:4 (20:80), the system successfully achieved zero-miss detection (FNR = 0%), while maintaining improved classification stability, demonstrating high sensitivity and strong learning capability for defect features.

Ablation studies further validated the importance of each design component. The SSIM-based sample selection strategy and triplet loss optimization were key to reducing the missed detection rate, while high defect rate training strategy and image transformation contributed significantly to improving model generalization and stability.

Moreover, the proposed ResNet-SE-CBAM Siamese system was benchmarked against YOLOv7 and YOLOv12 models under both 1:1 and 1:2 data ratios. While YOLOv12-Nano and YOLOv12-Small achieved a higher accuracy (90%) under the 1:1 setting, their FNRs remained relatively high at 18%. In contrast, our method achieved a lower FNR (14%) and better balanced performance, with an MCC of 0.72. Under the 1:2 condition, our system significantly outperformed all baselines, achieving 94% accuracy, an FNR of only 2%, and the highest MCC (0.88). These results demonstrate the proposed method’s superior stability and reliability for imbalanced, small-sample defect detection scenarios.

In conclusion, the proposed approach demonstrated stable and efficient defect detection performance under extremely limited and highly imbalanced data scenarios, showcasing strong potential and feasibility for real-world production line applications. Future work will focus on model lightweighting to improve inference speed and extending the system to handle multi-class and multi-defect-type scenarios, further enhancing adaptability and generalization in practical industrial environments.

While the proposed approach demonstrates strong performance and deployment feasibility, several limitations remain to be addressed:

- The proposed system is designed for few-shot defect detection and may underperform on production lines that lack high-quality golden samples. Our experiments show that both the quantity and diversity of golden samples significantly impact classification results. Future work may explore active learning or semi-supervised approaches to expand the golden sample set.
- The ResNet-SE-CBAM architecture, while effective, is relatively resource-intensive. It may pose deployment challenges on resource-constrained edge devices such as embedded SoCs or FPGAs. Model compression or architectural simplification will be explored for broader edge compatibility.
- Our model is currently structured for binary classification (defective vs. normal) and cannot identify specific defect types or locations from training samples. Future extensions may integrate semantic segmentation or multi-class classification to enhance interpretability and diagnostic utility.
- Although good results have been observed on both resistor and capacitor components, further evaluation is needed to assess generalization to unseen PCB component types and defect categories.

## Figures and Tables

**Table 1:** Dataset composition.

Category	Number of Images	Proportion (%)
Normal Samples (Good)	900	85.7%
Defective Samples	150	14.3%
Total	1050	100%

**Table 2:** Experimental configurations.

Number of Good Samples	Number of Defective Samples	Ratio (Defective/Good)
20	20	1:1
20	40	2:1
20	60	3:1
20	80	4:1
20	100	5:1

**Table 3:** Performance comparison of different Siamese architectures.

Siamese Model	ACC	FNR	MCC
CNN Siamese	89%	4%	0.79
SE Siamese	86%	2%	0.74
ResNet Siamese	90%	2%	0.81
ResNet-SE Siamese	90%	0%	0.82
ResNet-CBAM Siamese	86%	6%	0.73
ResNet-CA Siamese	92%	0%	0.85
ResNet-SE-CBAM Siamese	94%	2%	0.88
ResNet-SE-CBAM-CA Siamese	94%	4%	0.88

**Table 4:** Performance comparison of different classifiers.

Classifier	ACC	FNR	MCC
KNN	94%	2%	0.88
SVM	93%	2%	0.79
XGBoost	84%	0%	0.72
MLP	93%	2%	0.83

**Table 5:** Model performance under different defect-to-good sample ratios.

Pass/NG Ratio (K = 3)	ACC	FNR	MCC
20:20 (1:1)	82%	14%	0.72
20:40 (1:2)	94%	2%	0.88
20:60 (1:3)	92%	2%	0.84
20:80 (1:4)	86%	0%	0.75
20:100 (1:5)	83%	0%	0.70

**Table 6:** Performance comparison between the proposed method and YOLO series models.

Model	Ratio	Accuracy	FNR	MCC
ResNet-SE-CBAM Siamese + KNN (our)	1:1	82%	14%	0.72
YOLOv7-tiny	1:1	74%	6%	0.52
YOLOv7	1:1	63%	42%	0.26
YOLOv12-Nano	1:1	90%	18%	0.80
YOLOv12-Small	1:1	90%	18%	0.80
ResNet-SE-CBAM Siamese + KNN (our)	1:2	94%	2%	0.88
YOLOv12-Nano	1:2	91%	18%	0.83
YOLOv12-Small	1:2	91%	18%	0.83

**Table 7:** Performance comparison between the original and GAN-generated datasets.

Dataset (Pass/NG)	Accuracy	FNR	MCC
Trained with original dataset (20:40)	94%	2%	0.88
Trained with GAN-generated dataset (20:240)	84%	10%	0.68

**Table 8:** Evaluation of resistor dataset for metric learning with pre-trained and zero-shot testing.

Model (with KNN, K = 3)	Mode	Ratio	Accuracy	FNR	MCC
ResNet-SE-CBAM Siamese	Zero-shot	Pre-trained 20:20	95%	10%	0.90
ResNet-SE-CBAM Siamese	Zero-shot	Pre-trained 20:40	95%	10%	0.90
CNN Siamese	Trained	20:40	85%	0%	0.81
ResNet Siamese	Trained	20:40	75%	10%	0.67
SE Siamese	Trained	20:40	85%	10%	0.78
ResNet-SE-CBAM Siamese	Trained	20:20	95%	10%	0.93
ResNet-SE-CBAM Siamese	Trained	20:40	90%	0%	0.87

## Data Availability

Data Availability Statement: All data and source code presented in this study are openly available at https://github.com/evan6007/ResNet-SE-CBAM_Siamese (accessed on 10 May 2025), under the MIT License.
